# Preliminary Characterization, Antioxidant and Hepatoprotective Activities of Polysaccharides from Taishan *Pinus massoniana* Pollen

**DOI:** 10.3390/molecules23020281

**Published:** 2018-01-30

**Authors:** Changming Zhou, Shaojie Yin, Zhongfang Yu, Yuxiang Feng, Kai Wei, Weiming Ma, Lijiang Ge, Zhengui Yan, Ruiliang Zhu

**Affiliations:** 1 College of Animal Science and Veterinary Medicine, Shandong Agricultural University, Tai’an 271018, China; zcm_srr2011@sdau.edu.cn (C.Z.); yinsj1989@163.com (S.Y.); 18763831291@163.com (Z.Y.); VonYuhasing@163.com (Y.F.); weikaisdau@163.com (K.W.); mawm@sdau.edu.cn (W.M.); glj@sdau.edu.cn (L.G.); 2 College of Veterinary Medicine, Yangzhou University, 12 East Wenhui Road, Yangzhou 225009, China; 3 Research Center for Animal Disease Control Engineering Shandong Province, Shandong Agricultural University, Tai’an 271018, China

**Keywords:** Taishan *Pinus massoniana* pollen, polysaccharides, characterization, antioxidant activity, hepatoprotective effect

## Abstract

The objectives of the present study were to characterize the chemical composition, antioxidant activity and hepatoprotective effect of the polysaccharides from Taishan *Pinus massoniana* pollen (TPPPS). HPLC analysis showed that TPPPS was an acidic heteropolysaccharide with glucose and arabinose as the main component monosaccharides (79.6%, molar percentage). Fourier transform-infrared spectroscopy (FT-IR) analysis indicated that the spectra of TPPPS displayed infrared absorption peaks characteristic of polysaccharides. In in vitro assays TPPPS exhibited different degrees of dose-dependent antioxidant activities , and this was further verified by suppression of CCl_4_-induced oxidative stress in the liver with three tested doses of TPPPS (100, 200, and 400 mg/kg bw) in rats. Pretreatment with TPPPS significantly decreased the levels of alanine aminotransferase (AST), aspartate aminotransferase (ALT), alkaline phosphatase (ALP), lactic dehydrogenase (LDH) and malondialdehyde (MDA) against CCl_4_ injuries, and elevated the activities of superoxide dismutase (SOD) as well as glutathione peroxidase (GSH-Px). Histopathological observation further confirmed that TPPPS could protect the liver tissues from CCl_4_-induced histological alternation. These results suggest that TPPPS has strong antioxidant activities and significant protective effect against acute hepatotoxicity induced by CCl_4_. The hepatoprotective effect may partly be related to its free radical scavenging effect, increasing antioxidant activity and inhibiting lipid peroxidation.

## 1. Introduction

Reactive oxygen species (ROS) generated in aerobic organisms during the respiration process, including free radicals such as superoxide anion (O_2_^•−^), hydroxyl radical (OH^•^) and hydrogen peroxide (H_2_O_2_), play important roles in degenerative and pathological processes such as cancer, aging, inflammation and fibrosis [1,2,3]. Mammalian cells constantly exposed to excessive ROS may suffer detrimental effects such as lipid peroxidation of cellular membranes, enzyme inactivation, DNA breakage, alteration of lipid–protein interaction and eventually the promotion of mutations that initiate tumor progression [4]. It has also been found that ROS are involved in various acute and chronic liver diseases [5]. Although the synthetic antioxidants such as propyl gallate and butylated hydroxytoluene could effectively inhibit the oxidative damage, their potential toxicity in human body has been reported and is of concern [6]. In recent years, many natural polysaccharides, which are widely found in animals, plants and microorganisms, have been demonstrated to play an important role as free radical scavengers in the prevention of oxidative damage in living organism, and can be exploited as novel potential antioxidants to prevent the damage of ROS [7,8,9]. Therefore, discovery and assessment of natural polysaccharides as new functional antioxidant medicines have become a hot research field.

*Pinus massoniana* pollen, a traditional food supplement and medicine in China, is continually reported to have a wide range of health benefits including alleviating fatigue, delaying apolexis, and treating disease [10]. These beneficial effects are attributed to its variety of chemical ingredients including proteins, vitamins, enzymes and coenzymes, fats, flavonoids, nucleic acids, monosaccharides, polysaccharides, phospholipids, and other nutrients [11]. Recent studies have demonstrated that Masson pine pollen had a hepatoprotective effect which was related to its antioxidant properties [12]. Taishan *Pinus massoniana* pollen polysaccharide (TPPPS), is extracted from *Pinus massoniana* pollen collected from Mount Tai (Chinese name: Taishan). Our previous studies have confirmed that TPPPS could enhance immunological functions in mice, rabbits and chickens, and significantly improve the immune response of the subunit vaccine [13,14,15,16]. However, there is relatively little information pertaining to the antioxidant and hepatoprotective activities of TPPPS. Therefore, the aim of the present study was firstly to characterize the chemical composition of TPPPS and its antioxidant activity in vitro and secondly, to explore the in vivo protective effect against carbon tetrachloride (CCl_4_)-induced hepatic damage in rat.

## 2. Results

### 2.1. Physicochemical Properties of TPPPS

The crude TPPPS were obtained from dry Taishan *Pinus massoniana* pollen using hot water extraction and ethanol precipitation. After deproteinization, dialysis and purification by gel-filtration chromatography, the yield of TPPPS was 4.28% of the starting material weight. The total carbohydrate content in TPPPS was 94.7%, as determined by the phenol–sulfuric acid method. As determined by the sulfuric acid-carbazole method, the content of uronic acid in TPPPS was 12.49%. Purified TPPPS appeared as a white powder, with no absorption at 260 nm in the UV spectrum, indicating the absence of nucleic acids. In addition, the Coomassie Brilliant Blue method showed that the protein content in TPPPS was 1.92%, suggesting that TPPPS might be a protein-bound polysaccharide.

**Figure 1:**
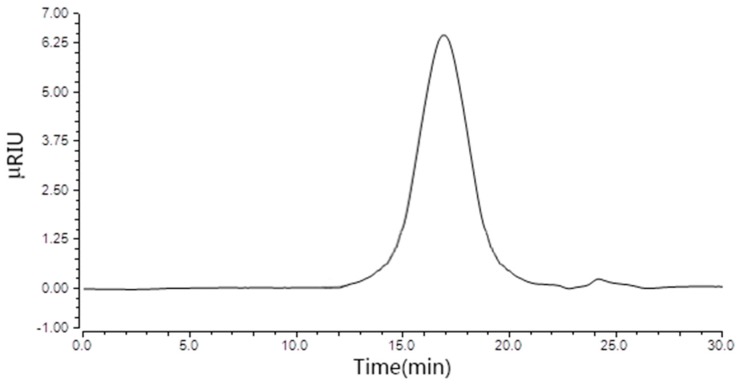
HPGPC profile of TPPPS.

**Figure 2:**
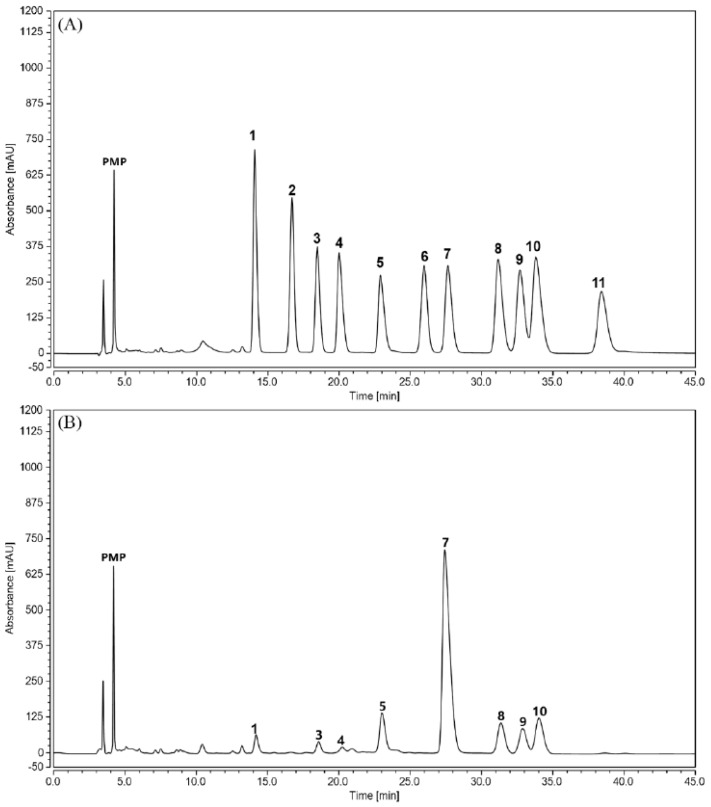
The HPLC chromatograms of PMP derivatives of component monosaccharides released from 11 standard monosaccharides (**A**) and TPPPS (**B**). Peaks: (1) mannose, (2) glucosamine, (3) rhamnose, (4) gluconic acid, (5) galacturonic acid, (6) galactosamine, (7) glucose, (8) galactose, (9) xylose, (10) arabinose, (11) fucose (internal standard).

As shown in Figure 1, TPPPS was eluted as a single symmetrical peak in the high performance gel permeation chromatography (HPGPC) chromatogram, indicating that it was a homogeneous polysaccharide. The monosaccharide composition and content in TPPPS were determined using a high-performance liquid chromatography (HPLC) method (Figure 2). Following derivatization with 1-phenyl-3-methyl-5-pyrazolone (PMP) eleven kinds of standard monosaccharides could be separated well through the column within 45 min. As shown in Figure 2B, TPPPS was composed of mannose, rhamnose, gluconic acid, galacturonic acid, glucose, galactose, xylose and arabinose, and their corresponding mole percentages were 1.76%, 1.67%, 0.78%, 5.39%, 69.17%, 5.18%, 5.63% and 10.43% of all the total monosaccharides, respectively.

### 2.2. FT-IR Spectrum of TPPPS

**Figure 3:**
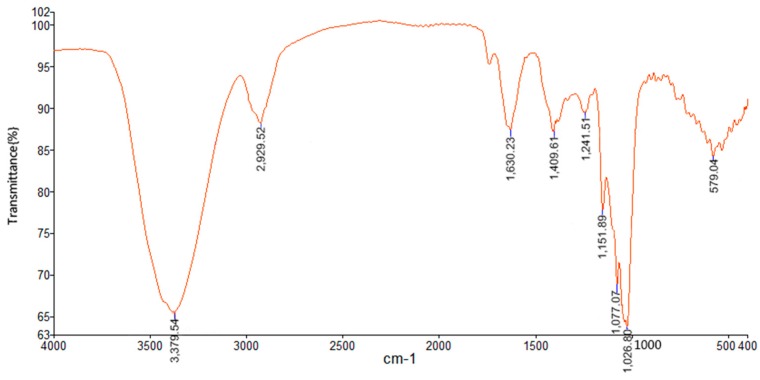
FT-IR spectrum of TPPPS in the frequency range of 4000–400 cm^−1^.

To characterize the TPPPS composition more precisely, the characteristic FT-IR spectrum was recorded in the wavelength range of 4000–400 cm^−1^ (Figure 3). Specifically, the strong and broad absorption peak at 3379 cm^−1^ is a characteristic stretching vibration of O-H, and the weak peak at 2929 cm^−1^ was caused by the stretching vibration of the C–H bonds. As reported, the characteristic absorption peak at 1026 and 1077 cm^−1^ was ascribed to the bending vibration of C–OH side groups and C–O–C glycosidic bond, indicating that there existed pyranose unit in TPPPS [17,18]. In addition, the stretching absorption peak at 1630 cm^−1^ along with the peak at 1409 cm^−1^ indicated the presence of carboxylic group (COO^−^), which conforms to the presence of uronic acids detected by the HPLC method [19,20].

### 2.3. In Vitro Antioxidant Activity of TPPPS

**Figure 4:**
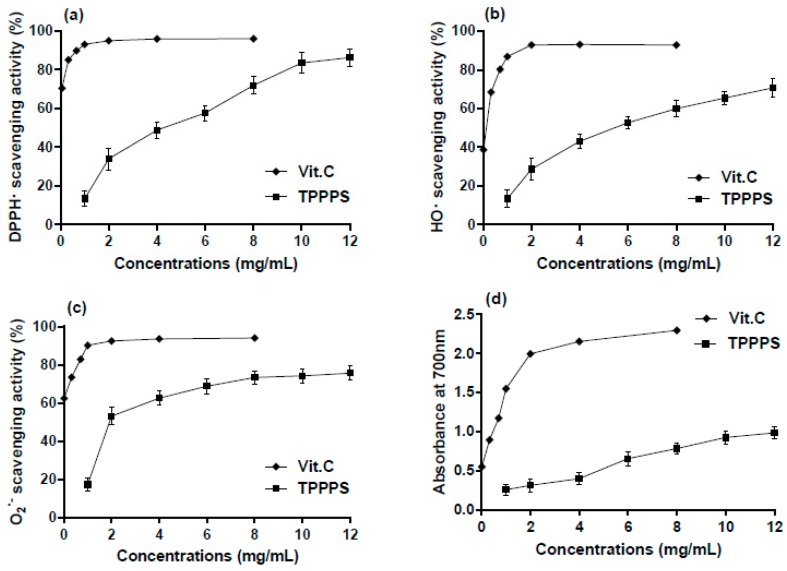
In vitro antioxidant activities of TPPPS determined using DPPH^•^ scavenging assay (**a**); HO^•^ scavenging assay (**b**); O_2_^•^^−^ scavenging assay (**c**) and ferric reducing powder assay (**d**). Vit. C was used as positive control.

In this study, the in vitro antioxidant capacity of TPPPS was estimated with DPPH^•^, O_2_^•^^−^ and HO^•^ and reducing power systems (Figure 4). 

As shown in Figure 4a, the DPPH^•^-scavenging activities of TPPPS were increased along with the increase of concentration. At the concentration of 12 mg/mL the DPPH^•^-scavenging activity of TPPPS (86.31%) was close to that of the reference substance vitamin C (Vit. C, 94.96%). Similarly, TPPPS also exhibited obvious scavenging activity against HO^•^ in the range of 13.43–70.71% at the concentration of 1–12 mg/mL (Figure 4b). In addition, TPPPS was also observed to possess the high ability to scavenge O_2_^•−^ (Figure 4c). The scavenging ratio was 14.47% at 1 mg/mL of TPPPS, and reached 75.74% when the concentration increased to 12 mg/mL. Furthermore, the antioxidant capacity of TPPPS was assessed by means of reducing power. As expected, in the broad range of 1–12 mg/mL, the phenomenon of concentration-dependence was still obvious (Figure 4d), and the reducing power (increased absorbance at 700 nm) of TPPPS ranged from 0.20 to 0.68 in a concentration-dependent manner.

### 2.4. The Toxicity of TPPPS

The oral toxicity of TPPPS was determined in male Wistar rats. There was no mortality observed in all the groups. In addition, all treatments caused no significant effects on food and water intake, general signs, pathological changes of organs, hematological and biochemical parameters (data not shown). Thus, the present study confirmed that TPPPS does not cause any apparent toxicity in a rat model.

### 2.5. Effects of TPPPS on Body Weight, Liver Weight and Hepatosomatic Index (HI)

The effects of TPPPS on the body weight, liver weight and HI for experimental rats are summarized in Table 1. For the CCl_4_ (model control) group, significant increases in body weight, liver weight and HI , respectively, were observed compared to the normal control group (*p* < 0.05, *p* < 0.01). However, the CCl_4_-induced increases in the liver weight and HI could be decreased by the pretreatment with TPPPS at the higher doses of 200 and 400 mg/kg bw relative to the CCl_4_ group (*p* < 0.05, *p* < 0.01, respectively). A similar decreasing effects were also observed on both the indexes with the pretreatment of the positive control Bifendate Pills (BP) at 100 mg/kg bw. The results indicated that the administration of TPPPS to rats may generate a comparable preventive effect against CCl_4_-induced liver damage as BP.

### 2.6. Effects of TPPPS on Activities of ALT, AST, ALP and LDH in Serum

**Figure 5:**
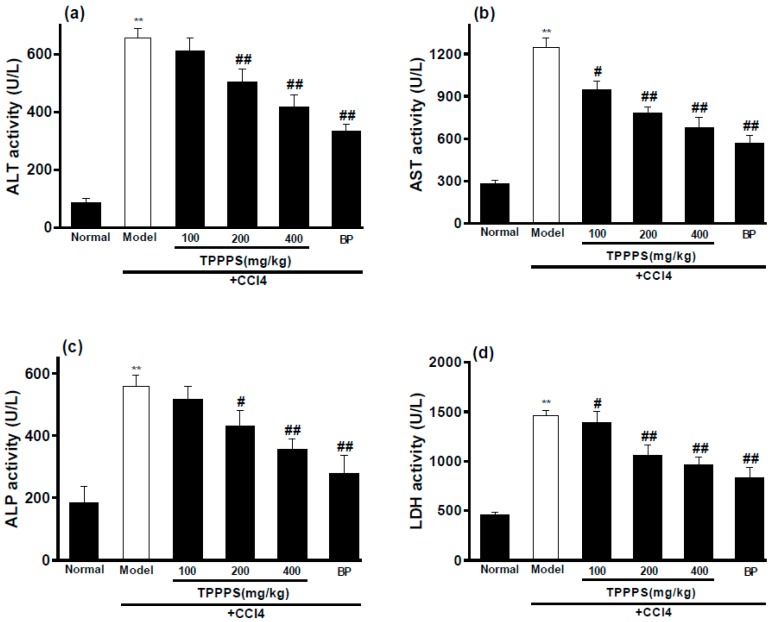
Effects of TPPPS on the enzymic activities of ALT (**a**); AST (**b**); ALP(**c**) and LDH (**d**) in serum after treatment with CCl4 in rats. Values are expressed as means ± SD for 6 rats in each group. ** *p* < 0.01, compared to the normal control group. ^#^ *p* < 0.05, ^##^ *p* < 0.01, compared to the model control group. Bifendate Pills (BP) was used as positive control.

The protective effects of TPPPS on serum ALT, AST, ALP and LDH activities are presented in Figure 5. The model control group exhibited significantly higher levels of ALT, AST, ALP and LDH in serum compared with the normal control group (*p* < 0.01), reflecting the tissue damages in the liver. As expected, the administration of TPPPS prior to the CCl_4_ intoxication effectively decreased the CCl_4_-induced elevations of serum enzymes (ALT, AST, ALP and LDH) in a dose-dependent manner. Specifically, at dosages of 200 and 400 mg/kg bw of TPPPS, the activities of ALT, AST, ALP and LDH were significantly decreased relative to the model control group (*p* < 0.05 or *p* < 0.01), which were close to that of the positive control BP at 100 mg/kg bw.

### 2.7. Effects of TPPPS on the Levels of MDA, SOD and GSH-Px in Hepatic Tissue

As shown in Table 2, there was a significant increase in MDA level and decrease in the activities of SOD as well as GSH-Px in model control group compared with the normal control group (*p* < 0.01), suggesting stronger oxidative stress and lipid peroxidation in liver tissue. However, the administration of TPPPS decreased the level of MDA, whereas enhanced the activities of SOD and GSH-Px in a dosage-dependent manner. Especially, at the dosage of 200 and 400 mg/kg bw, the MDA level in hepatic tissue was significantly reduced, whereas the SOD and GSH-Px activities were significantly elevated in comparison with the model control group (*p* < 0.05 or *p* < 0.01). The protective effects of TPPPS were nearly as good as BP at 100 mg/kg bw.

### 2.8. Histopathological Examination of Liver Tissues

**Figure 6:**
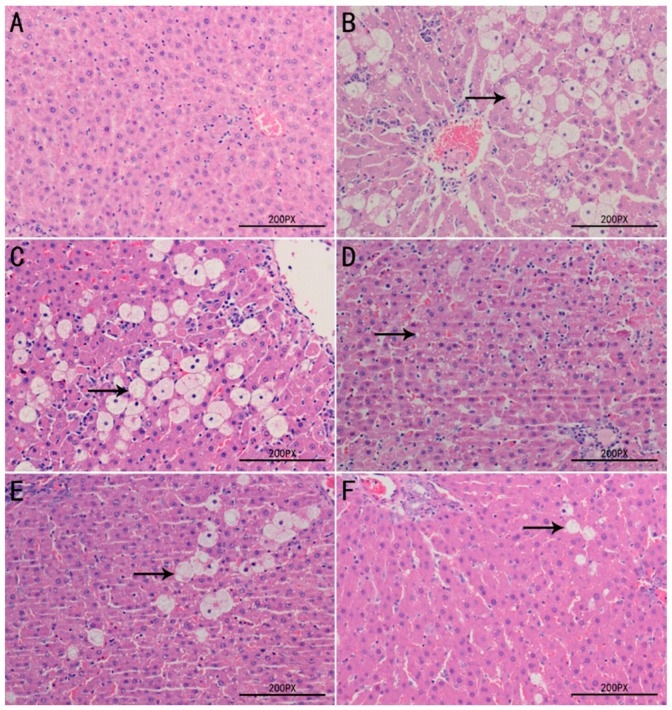
Effects of TPPPS on liver histopathological changes of rats using H & E staining (×200). Liver tissues of normal control group (**A**); Vehicle + CCl4 group (**B**); 100 mg/kg bw TPPPS + CCl4 group (**C**); 200 mg/kg bw TPPPS + CCl4 group (**D**); 400 mg/kg bw TPPPS + CCl4 group (**E**); 100 mg/kg bw BP + CCl4 group (**F**). Arrow indicate hepatocyte ballooning.

The protective effects exerted by TPPPS against CCl_4_–induced hepatotoxicity were further confirmed by histological assessment (Figure 6). The histological feature of the liver sections of the normal control group showed that hepatocytes were well-preserved and uniform cytoplasm, and prominent nuclei and central veins were obvious visible (Figure 6A). Compared with the normal control group, the sections of model control group revealed serious pathological changes characterized by hepatocellular degeneration and necrosis around the central vein, cytoplasmic vacuolation, inflammatory cell infiltration and loss of cellular boundaries (Figure 6B). However, as demonstrated in Figure 6C–F, the pre-administration with TPPPS remarkably ameliorated the hepatic histopathological lesions induced by CCl_4_ in a dose-dependent manner, and this was in good agreement with the results of biochemical assays.

## 3. Discussion

In recent years, the potential of using antioxidants, especially the natural antioxidants, in preventing and curing diseases has attracted enormous interest. Antioxidants may have a protective effect in preventing cancer, aging, heart and liver disease or lessening their severity [21]. This protective effect is attributed to their ability to scavenge ROS, which are generated during the oxidative stress. Thus, the usefulness of antioxidants in protecting cellular components against oxidative damage is well established [22]. In the present study, it was reported for the first time the antioxidant and hepatoprotective activities of TPPPS.

In our study, the TPPPS have been successfully extracted from pulverized *Pinus massoniana* pollen collected from the Taishan region. Furthermore, TPPPS was characterized as an acidic heteropolysaccharide, rich in glucose (69.17%) and arabinose (10.43%) among all the monosaccharides. The infrared spectrum of TPPPS showed characteristic absorption peaks of polysaccharides. In addition, the in vitro assays to measure scavenging of DPPH^•^, O_2_^•−^ and HO^•^ and ferric-reducing power showed that TPPPS had a concentration-dependent antioxidant effect. The data presented here indicate that TPPPS has good antioxidant capacity and the potential to be explored as a strong antioxidant.

Many studies have showed that some natural products with antioxidant activity also have hepatoprotective activity [23]. The hepatic injury model is commonly used to detect the hepatoprotective activity of drugs in vivo [24]. CCl_4_-induced liver damage is the best characterized system of xenobiotic-induced hepatotoxicity and a commonly used model for the hepatoprotective drugs screening [25,26]. The mechanism of liver injury induced by CCl_4_ may be associated with the severity of lipid peroxidation and the depletion of antioxidant status which is caused by damage of the cell membrane and the organelles of the hepatocyte [27,28,29]. It has been reported that administration of CCl_4_ in rats caused the increases in ALT, AST as well as ALP activities, lipid peroxidation products, and hepatocellular necrosis, and a decrease in antioxidative enzymes [30,31]. In the present study, administering intraperitoneally (ip) with 2.0 mL/kg bw of 50% CC1_4_ caused dramatic elevations of ALT, AST, ALP and LDH activities in serum and serious histopathological changes in liver tissue, indicating hepatotoxicity induced by administration of CC1_4_ in rats. However, the pretreatment of TPPPS, especially at the higher dosages of 200 and 400 mg/kg bw, significantly decreased the activities of ALT, AST, ALP and LDH, suggesting that TPPPS may effectively protect hepatocytes against the toxic effects of CCl_4_. These results indicated that TPPPS possess the potent hepatoprotective activity in vivo.

In order to more clarify the mechanisms of hepatoprotective activity of TPPPS, the effect on hepatic antioxidant defense system was explored. It is well known that SOD and GSH-Px are two major enzymes in antioxidant-defense system of organism, and MDA is the main product of lipid peroxidation and used as an indicator of reflecting oxidative damage and antioxidation [32,33]. The results of the present study showed that administration of TPPPS significantly elevated the enzymatic activities SOD and GSH-Px, and inhibited the formation of MDA in liver tissues compared to the model control group, especially at the dosages of 200 and 400 mg/kg bw, suggesting that TPPPS possesses in vivo antioxidative activities. Histological changes in the liver can directly reflect the degree of liver injury and repair. In the present study, in agreement with the results of the biochemical parameters assay in serum and liver tissues, administration of TPPPS reduced the histopathological alteration induced by CCl_4_. This may be due to preventing the toxic chemical reactions from forming highly reactive radicals induced by CCl_4_.

## 4. Materials and Methods

### 4.1. Materials and Reagents

The Taishan *Pinus massoniana* pollen was collected from the Taishan Region in Shandong Province, China. The voucher specimens were deposited in the College of Veterinary Medicine & Animal Science of Shandong Agricultural University. Monosaccharide standards, 1-phenyl-3-methyl-5-pyrazolonde (PMP), 2,2-diphenyl-1-picrylhydrazyl (DPPH), ascorbic acid (Vit. C) were purchased from Sigma Chemical Co. (St. Louis, MO, USA). CCl_4_ was purchased from Tianjin Fengchuan Chemical Reagent Science and Technology Co., Ltd. (Tianjin, China). Bifendate Pills (BP) was purchased from Beijing Union Pharmaceutical Factory (Beijing, China). Assay kits for malondialdehyde (MDA), superoxide dismutase (SOD) and glutathione peroxidase (GSH-Px) were purchased from Nanjing Jiancheng Bioengineering Institute (Nanjing, China). All other chemicals and reagents were of analytical grade.

### 4.2. Animals

Male Wistar rats weighing 180–200 g were purchased from the Experiment Animal Center of Academy of Military Medical Sciences (Beijing, China). The rats were housed under ideal laboratory conditions (12 h light/12 h darkness cycle, 45–55% relative humidity and temperature 23–25 °C), maintained on standard pellet diet and water ad libitum throughout the experimental period. All procedures involving animals were performed in accordance with the guidelines for the care and use of laboratory animals, as adopted and promulgated by Shandong Agricultural University Animal Care and Use Committee (Permit number: SDAUA-2014-010).

### 4.3. Preparation of TPPPS

Dry Taishan *Pinus massoniana* pollen collected from the Taishan Region in China, was passed through a 260 mesh sieve, and pulverized by an Ultra-Micro Pulverizer (Zhengxin Science and Technology Co., Ltd., Taian, China). TPPPS was extracted in our laboratory according to the method described by Wei et al. [13] with some modifications. Briefly, crude TPPPS were extracted by water decoction and one-step ethanol precipitation, in which ethanol was added to the decoction to obtain ethanol concentration of 80% (*v*/*v*). The crude TPPPS was again purified using Sevag’s method to eliminate protein [34], active carbon absorption to remove the pigment and through a Sephadex G-75 column eluted with 0.15 M NaCl solution. Then the fraction with the highest peak on the distribution curve was dialyzed, concentrated, and freeze-dried to obtain the purified TPPPS. The yield of TPPPS were calculated.

### 4.4. Preliminary Characterization of TPPPS

The total carbohydrate content of TPPPS was determined by a modified phenol-sulfuric acid colorimetric method with glucose as a standard [35]. Uronic acid contents were estimated using a modified sulfuric acid-carbazole method with gluconic acid as the standard [36]. In addition, proteins in the polysaccharides were determined by the method using Coomassie Brilliant Blue G-250 with bovine serum albumin as the standard [37]. The homogeneity of TPPPS were determined by the HPGPC method described by Yan et al. [38]. The monosaccharide components of the TPPPS were analyzed by reverse-phase HPLC using the PMP derivatization procedure with some modification [20]. FT-IR spectroscopy was employed to detect the main functional groups in TPPPS. The FT-IR spectrum was recorded on a Nicolet 6700 FT-IR spectrometer (Thermo Fisher Scientific Inc., Madison, MA, USA) in the wavenumber region of 4000–400 cm^−1^. The TPPPS sample was mixed and grounded with potassium bromide. The spectrum was obtained from 120 scans at 4 cm^−1^.

### 4.5. Determination of Antioxidant Activity In Vitro

The antioxidant activities of TPPPS were demonstrated by ferric reducing powder and three radicals scavenging methods reported previously, including DPPH^•^, O_2_^•−^ and HO^•^. Each test was performed in triplicates.

#### 4.5.1. Scavenging Activity on DPPH^•^

The assay was performed as described by Wang et al. [39] with slight modification. In brief, 1.0 mL of TPPPS solution at various concentrations (1–12 mg/mL) was mixed with 3.0 mL of 0.1 mM DPPH in aqueous methanol. The mixed solutions were measured at absorbance of 517 nm after incubation for 30 min in the dark at room temperature. The DPPH^•^ scavenging activity (%) was calculated with the following formula: Scavenging activity (%) = [1 − (As − A_b_)/A_0_] × 100, where A_0_ was the absorbance without sample, As was the absorbance with sample, and A_b_ was the absorbance of ground color. Vit. C was used as positive control.

#### 4.5.2. Scavenging Activity on HO^•^

The scavenging activity of TPPPS on HO^•^ was measured according to the method described by Huang et al. [40]. In brief, 1.0 mL TPPPS solution at various concentrations (1–12 mg/mL) were incubated with FeSO_4_ (1 mL, 4 mM), salicylic acid-ethanol (1 mL, 6 mM), and H_2_O_2_ (1 mL, 2 mM) at 37 °C for 60 min, and then the absorbance at 510 nm was measured. The scavenging activity was calculated by the following equation: Scavenging activity (%) = [1 − (A_1_ − A_2_)/A_0_] × 100, where A_0_ was the absorbance of the control, A_1_ was the absorbance of the sample and A_2_ was the absorbance without H_2_O_2_. Vit. C was used as positive control.

#### 4.5.3. Scavenging Activity on O_2_^•−^

The activities of TPPPS to scavenge O_2_^•−^ were determined using the method previously reported [41]. 1.0 mL nitroblue tetrazolium (NBT), 1.0 mL reduced nicotinamide adenine dinucleotide (NADH), 1.0 mL TPPPS solution at various concentrations (1–12 mg/mL) and 0.4 mL phenazine methosulfate (PMS) were successively added into test tube, then the reaction mixture was incubated at room temperature for 5 min and the absorbance of the mixture solution was determined at 560 nm against blank (mixture without PMS was used as blank). The scavenging capability of TPPPS was calculated according to the following formula: Scavenging activity (%) = [1 − (Abs. of sample − Abs. of blank)/Abs. of control] × 100. Vit. C was used as positive control.

#### 4.5.4. Measurement of Reducing Power

The reducing power of TPPPS was measured according to the method of Tian et al. [19]. Briefly, 2.5 mL of 0.2 M phosphate buffer (pH 6.6) and 2.5 mL of 1% (*w*/*v*) K_3_Fe(CN)_6_ solution were added to 1.0 mL of TPPPS solution at various concentrations (1–12 mg/mL). The mixture was incubated at 50 °C for 20 min, and then 2.5 mL 10% (*w*/*v*) trichloroacetic acid (TCA) solution was added, and the mixture was further centrifuged at 3000 r/min for 10 min. The absorbance at 700 nm was measured immediately. An enhanced absorbance of the reaction mixture indicated a high reducing power. Vit. C was used as a positive control.

### 4.6. Determination of the Toxicity of TPPPS

Male Wistar rats weighing 180–200 g was randomly divided into seven groups of five animals each. The normal control group received saline (10 mL/kg, oral) and other groups received 50, 100, 200, 400, 800 and 1600 mg/kg of TPPPS dispersion in saline. Food and water intake, general signs, and mortality were observed continuously for 14 days. Blood samples and pathology of organs were evaluated at day 14.

### 4.7. Determination of Hepatoprotective Activity In Vivo

#### 4.7.1. CCl_4_-induced Hepatotoxicity Experiment

After a 7-day acclimatization period, a total of 36 rats were randomly divided into six groups of six rats each. For the normal and model control groups, rats were given a single dose of physiological saline (2.0 mL) once daily intragastrically (ig). For the BP (positive) group, rats received 100 mg/kg bw of reference drug BP (2.0 mL, ig) once daily. For the TPPPS-treated groups, rats were given 100, 200, or 400 mg/kg bw of TPPPS (2.0 mL, ig) once daily, respectively. All the administrations were conducted at between sixteen and seventeen o’clock for 14 consecutive days. On the 15th day, all the rats except the normal control group were given a 50% CCl_4_/peanut oil mixture (*v*/*v*, 2.0 mL/kg bw) intraperitoneally (ip) to induce hepatic injury, while the normal control group received peanut oil alone. After 2 h, all the rats were fasted with only water provided for 24 h. And then all of the rats were weighed and anesthetized by pentobarbital sodium (40 mg/kg bw, ip). Blood samples were immediately collected from the heart, centrifuged to obtain sera, and stored at −70 °C for further biochemical analysis. Livers were immediately taken out and weighted after rinsing by ice-cold physiological saline and then stored at −80 °C before use. Hepatosomatic index (HI) was calculated according to the following formula: HI = liver weight/body weight × 100%.

#### 4.7.2. Serum Biochemical Indicators Assay

The activities of ALT, AST, ALP and LDH were determined with an Automatic Biochemical Analyzer (AU2700, Olympus, Kanagawa, Japan) using diagnostic reagent kits. All the experiments were conducted in triplicates, and the average counts were obtained from each individual sample.

#### 4.7.3. Antioxidative Indicators Assay of Liver Homogenate

The liver samples were homogenized in 9-fold volume cold physiological saline, and then the homogenates were centrifuged at 1500× *g* for 10 min. The clear supernatant fraction was used for SOD, GSH-Px and MDA analysis with the kits according to the instructions. The protein concentration in homogenates was measured by the method of Coomassie Brilliant Blue with bovine serum albumin as a standard [37]. 

#### 4.7.4. Histopathological Examination of Liver

Some parts of liver tissues were fixed with a 4% paraformaldehyde solution at 4 °C for 24 h. Fixed liver tissues were embedded in paraffin wax, cut into sections (~5 μm) and stained with hematoxylin-eosin (H & E) solution. The slides were observed under an Olympus^®^ light microscope (Olympus Corporation, Tokyo, Japan) and photographed.

### 4.8. Statistical Analysis

The data were expressed as means ± standard deviation (SD) and evaluated by one-way analysis of variance followed by the LSD-test using the SPSS 18.0 system (SPSS Inc., Chicago, IL, USA). The significant difference was considered at *p* < 0.05, and extremely significant difference was considered at *p* < 0.01.

## 5. Conclusions

In this study, TPPPS displayed overall antioxidant activity against in vitro oxidative stress as well as in vivo CCl_4_-induced oxidative hepatotoxicity in rat. The hepatoprotective activity of TPPPS may, at least partly, be due to its free radical-scavenging activity, enhancing the antioxidant capacity of the liver. Our findings provide a base for further exploitation of TPPPS as a novel hepatoprotective pharmaceutical ingredient. Further work on the structure, function and mechanisms of action is in progress.

## Figures and Tables

**Table 1:** Effects of TPPPS on body weight, liver weight and HI of CCl_4_-intoxicated rats.

Treatments	Doses	Body Weight (g)	Liver Weight (g)	HI (%)
Normal	-	285 ± 17	13.2 ± 2.7	4.62 ± 0.48
CCl_4_	-	317 ± 15 *	18.3 ± 2.4 **	5.77 ± 0.23 **
CCl_4_ + TPPPS	100 mg/kg	305 ± 18	16.4 ± 1.9	5.36 ± 0.29 ^#^
CCl_4_ + TPPPS	200 mg/kg	299 ± 17	14.8 ± 2.4 ^#^	4.95 ± 0.36 ^##^
CCl_4_ + TPPPS	400 mg/kg	300 ± 16	14.8 ± 2.6 ^#^	4.92 ± 0.22 ^##^
CCl_4_ + BP	100 mg/kg	299 ± 16	14.8 ± 1.9 ^#^	4.94 ± 0.56 ^##^

Values are means ± SD for six rats in each group. * *p* < 0.05, ** *p* < 0.01, compared to the normal control group. ^#^ *p* < 0.05, ^##^ *p* < 0.01, compared to the model control group.

**Table 2:** Effects of TPPPS on the levels of MDA, SOD and GSH-Px in hepatic tissue of CCL_4_-induced liver injury in rats.

Treatments	Dose	MDA (nmol/mg Protein)	SOD (U/mg Protein)	GSH-Px (U/mg Protein)
Normal	-	2.23 ± 0.31	117.88 ± 16.20	67.20 ± 4.35
CCl_4_	-	7.63 ± 1.17 **	45.58 ± 9.89 **	34.96 ± 3.76 **
CCl_4_ + TPPPS	100 mg/kg	5.99 ± 1.31 ^#^	55.95 ± 8.64 ^#^	39.51 ± 4.62
CCl_4_ + TPPPS	200 mg/kg	4.71 ± 0.92 ^#^	77.10 ± 5.34 ^##^	45.56 ± 5.14 ^#^
CCl_4_ + TPPPS	400 mg/kg	3.71 ± 0.48 ^##^	94.74 ± 7.10 ^##^	56.79 ± 8.36 ^##^
CCl_4_ + BP	100 mg/kg	4.40 ± 0.38 ^#^	79.27 ± 6.31 ^##^	50.88 ± 3.47 ^##^

Values are means ± SD for six rats in each group. ** *p* < 0.01, compared to the normal control group. ^#^ *p* < 0.05, ^##^ *p* < 0.01, compared to the model control group.
